# A freeze-and-thaw-induced fragment of the microtubule-associated protein tau in rat brain extracts: implications for the biochemical assessment of neurotoxicity

**DOI:** 10.1042/BSR20203980

**Published:** 2021-03-24

**Authors:** Israel C. Vasconcelos, Raquel M. Campos, Hanna K. Schwaemmle, Ana P. Masson, Gustavo D. Ferrari, Luciane C. Alberici, Vitor M. Faça, Norberto Garcia-Cairasco, Adriano Sebollela

**Affiliations:** 1 Department of Biochemistry and Immunology, Ribeirao Preto Medical School, University of São Paulo, SP 14049-900, Brazil; 2 Department 1, University Children's Hospital, University of Tübingen, Tübingen D-72074, Germany; 3 Department of Biomolecular Sciences, School of Pharmaceutical Sciences of Ribeirao Preto, University of São Paulo, SP 14040-903, Brazil; 4 Department of Physiology, Ribeirao Preto Medical School, University of São Paulo, SP 14049-900, Brazil

**Keywords:** Alzheimer's disease, disease models, neurodegeneration, tau protein, tauopathy, western blot

## Abstract

Tau is a microtubule-associated protein (MAP) responsible for controlling the stabilization of microtubules in neurons. Tau function is regulated by phosphorylation. However, in some neurological diseases Tau becomes aberrantly hyperphosphorylated, which contributes to the pathogenesis of neurological diseases, known as tauopathies. Western blotting (WB) has been widely employed to determine Tau levels in neurological disease models. However, Tau quantification by WB should be interpreted with care, as this approach has been recognized as prone to produce artifactual results if not properly performed. In the present study, our goal was to evaluate the influence of a freeze-and-thaw cycle, a common procedure preceding WB, to the integrity of Tau in brain homogenates from rats, 3xTg-AD mice and human samples. Homogenates were prepared in ice-cold RIPA buffer supplemented with protease/phosphatase inhibitors. Immediately after centrifugation, an aliquot of the extracts was analyzed via WB to quantify total and phosphorylated Tau levels. The remaining aliquots of the same extracts were stored for at least 2 weeks at either −20 or −80°C and then subjected to WB. Extracts from rodent brains submitted to freeze-and-thaw presented a ∼25 kDa fragment immunoreactive to anti-Tau antibodies. An in-gel digestion followed by mass spectrometry (MS) analysis in excised bands revealed this ∼25 kDa species corresponds to a Tau fragment. Freeze-and-thaw-induced Tau proteolysis was detected even when extracts were stored at −80°C. This phenomenon was not observed in human samples at any storage condition tested. Based on these findings, we strongly recommend the use of fresh extracts of brain samples in molecular analysis of Tau levels in rodents.

## Introduction

Tau is a neuronal, microtubule-associated protein (MAP) responsible for controlling the stabilization of microtubules in neurons, thereby impacting the coordination of the axoplasmic transport of organelles, proteins, lipids, synaptic vesicles and other important cargos along the neuron [1,2]. Tau function is regulated by phosphorylation, but in some neurological diseases it is found aberrantly hyperphosphorylated [2,3]. In fact, the involvement of key phosphorylation sites in Tau dysfunction, such as serine (Ser) 396, has been well demonstrated [4–6]. Due to the strong correlation between high levels of hyperphosphorylated Tau, aggregation, microtubule destabilization and neuronal dysfunction/death, elevated hyperphosphorylated Tau levels have been assigned as a hallmark of several neuropathologies, collectively known as tauopathies [3,7–9]. In addition, it has been shown that increased production of Tau protein fragments, due to enzymatic cleavage, also correlates with neurodegeneration in cellular models [10] and, more importantly, with memory deficits in animal models [11].

Western blotting (WB) has been widely employed in the assessment of Tau protein levels (either total or hyperphosphorylated Tau), as well as in the analysis of Tau fragmentation in a number of studies [11–14]. However, quantification of Tau levels/cleavage by WB should be interpreted with care, as this approach has been recognized as a complex, multistep technique that requires case-to-case standardization and is subjected to produce artifactual results if not performed properly [15,16]. Of critical importance is the maintenance of the integrity of the protein of interest throughout processing, since unsuitable conditions for collection, storage, handling or experimental separation/detection may damage the target molecule. Thus, protein degradation could lead to conclusions that do not accurately reflect the biological phenomenon under investigation [15,17]. This issue is particularly relevant in studies comprising the analysis of a large set of samples, such as in the case of studies using rodents, in which storage by freezing is common.

The goal of the present study was to evaluate the influence of freeze-and-thaw prior to WB analysis on the integrity of the Tau protein in extracts from rat brains. We found that rat brain extracts submitted to a single freeze-and-thaw cycle presented fragmentation of Tau protein, as detected by the presence of an extra ∼25 kDa band simultaneously to the weakening of the bands corresponding to full-length Tau in samples prepared from both hippocampus and frontal cortex. Interestingly, Tau fragmentation was minor in brain extracts from 3xTg-AD mice, and absent from adult human brain extracts, suggesting that differences in Tau primary sequence between species may influence its liability to freeze-and thaw-triggered degradation. Based on this finding, we recommend that molecular analyses of Tau levels/fragmentation in rat brain extracts should be carried out only using fresh extracts, in order to produce results that accurately reflect the biological phenomenon under investigation.

## Methods

### Animals

The procedures were carried out using 12-month-old animals (*Rattus norvegicus*), both control Wistar (*n*=7) and Wistar Audiogenic Rat (WAR) (*n*=9) strains [18], were approved by the Ethics Committee on Animal Use of Ribeirão Preto Medical School (CEUA-FMRP), under the protocol #017/2014-1. Control Wistar rats were obtained from the Central Vivarium of the University of São Paulo (USP) at Ribeirão Preto and kept at 25°C, 12-h/12-h photoperiod and water and food *ad libitum*. WAR animals were obtained from the Vivarium of the Department of Physiology of the USP Ribeirão Preto Medical School and kept at the same conditions. The procedures carried out using 6–8-month-old animals (*Mus musculus*) transgenic mice 3xTg-AD (*n*=6) strain [19] were approved by the Ethics Committee on Animal Use of School of Pharmaceutical Sciences of Ribeirao Preto (CEUA-FCFRP), under the protocol 18.1.6.60.1. The 3xTg-AD mice strain was obtained from the Vivarium I of the School of Pharmaceutical Sciences of Ribeirao Preto and kept at 23°C, 12-h/12-h photoperiod and water and food *ad libitum*.

### *Ex vivo* human brain slices

The procedures carried out using *ex vivo* human brain slices [20] were approved by the Ribeirão Preto Medical School Ethics Committee, under the protocol HCRP #17578/15. Briefly, a fragment of human frontal cortex was surgically collected, sliced (200-μm-thick) and cultured for 2 days, as described in detail in [20,21].

### WB

Animals were anesthetized by inhalation of isoflurane (*BioChimico*, Brazil) and euthanized by decapitation. Cerebral tissue of rats and mice was collected, dissected and stored in 1.5 ml microtubes at −80°C. *Ex vivo* human brain slices were stored in 1.5 ml microtubes immediately prior to homogenization. Homogenates from dorsal portion of hippocampus (rat), frontal cortex (mice and rat) and *ex vivo* human brain slices were prepared according to Mendes et al. (2018), Fernandes et al. (2019) and Petry et al. (2014) [20–22]. Tissue fragments were homogenized in ice-cold RIPA buffer (50 mM Tris, 150 mM NaCl, 1 mM EDTA, 1% Triton X-100 and 0.1% SDS, pH 7.5) supplemented with protease inhibitor (1:100, Sigma) and phosphatase inhibitor (10 mM NaF, 10 mM Na_3_VO_4_) cocktails at the ratio of 10 µl per mg of tissue using an electric potter (Kimble Chase) in three cycles of 10 s each. Extracts were centrifuged for 10 min at 10000×***g*** and 4°C. Supernatant was transferred to new microtubes and aliquots were readily used for analysis of Tau protein by WB (fresh extract). The remaining aliquots of the same extracts were stored at −20 and −80°C for at least 2 weeks to 9 months (the precise storage time each extract underwent is presented in Supplementary Table S1) and then thawed just before WB (freeze/thaw extract). Preparation of samples for SDS/PAGE included addition of protein loading buffer (0.0625 M Tris-Cl, 2% SDS, 10% v/v glycerol, 0.1 M dithiothreitol, 0.01% Bromophenol Blue, pH 6.8) to extracts and boiling for 5 min at 100°C. Alternatively, some samples were diluted in SDS/PAGE loading buffer and boiled before storage at −20°C. The SDS/PAGE was carried out using 12% acrylamide/bis-acrylamide gels and a constant voltage of 90 V. For blotting, 0.45-µm nitrocellulose membranes (*GE Healthcare Life Sciences*) were utilized. Membranes were blocked in 5% nonfat dry milk/T-TBS (Tris-buffered saline: 20 mM Tris pH 7,5, 150 nM NaCl, 0,1% Tween 20) solution for 1 h at RT and subsequently incubated with primary antibody overnight at 4°C. Primary antibodies used for Tau assessment were anti-Tau phospho S396 (*Abcam*) or anti-Tau [E178] (*Abcam*), both 1:1000 in 5% BSA/T-TBS solution. After washing, membranes were incubated with secondary antibody ECL anti-rabbit IgG HRP (*Amersham*) at 1:3000 in 5% BSA/T-TBS for 1 h at RT. The membranes were revealed using *ECL Prime Western Blotting Detection Reagent* (*GE Healthcare Life Sciences*) and imaged in *ChemiDoc* imaging system (*Bio-Rad*) coupled to a digital system and software *ImageQuant*™ 3.5 (*GE Healthcare Life Sciences*). For β-actin probing, membranes were initially deblotted with stripping solution (10% SDS, 100 mM glicin, 0,1% NP 40, pH 2.5) for 30 min under vigorous shaking, washed in T-TBS solution then incubated with anti-β-actin (*EMD Millipore*) at 1:20000 in 3% BSA/T-TBS for 1 h at RT.

### In-gel digestion and LC-MS/MS analysis

Aliquots (250 µg total protein) of hippocampal extracts of WAR animals subjected to freeze/thaw were treated with 5 µl of dithiothreitol (*Bio-Rad*) 50 µg/μl, diluted in ammonium bicarbonate (*Sigma*) 100 mM, pH 7.8, for 30 min at 37°C. DTT-treated samples were then diluted in loading buffer, boiled for 5 min at 100°C, and alkylated in the dark for 30 min at 37°C with 1250 µg of iodoacetamide (*Sigma*) 50 µg/μl, diluted in ammonium bicarbonate 100 mM, pH 7.8. After sample separation by SDS/PAGE (carried out using 12% acrylamide/bis-acrylamide gels and a constant voltage of 90 V), a piece of approximately 30 mm^2^ of the unstained gel (per lane) was cut off using as a reference the 25 kDa standard band (pre-stained). Gel pieces corresponding to the fragment were further sliced to 1–4 mm^2^ pieces, washed and digested with trypsin (*Promega*) as described by Grassi et al. (2017). Tryptic peptides were successively extracted with 5% formic acid (*Merck*)/50% acetonitrile (*Merck*), and then 90% acetonitrile, and dried in a vacuum concentrator. The sample was re-suspended in 50 µl 50% acetonitrile/5% formic acid and centrifuged at 12000×***g*** for 15 min at 25°C. The supernatant was injected into an LC-MS/MS *Xevo TQS system* (*Waters*). Chromatographic separation was performed in a UPLC (I-class, *Waters*) using a C18 column (1.8 μm particle size, 100 Å pore size, 1 mm × 150 mm, Waters) in a linear gradient of 5–30% acetonitrile over 15 min at 100 μl/min in a formic acid:acetonitrile:water solvent system. Detection of Tau theoretical tryptic peptides was programmed in a scheduled multiple reaction monitoring method using three to five transitions per peptide and monitoring windows of 2 min centered on predicted retention time. Both method development and data analysis were conducted using *Skyline* software [23].

### Sequences alignment

The Tau sequences for rat (P19332), mouse (P10637) and human (P10636) were obtained from the Uniprot databank (https://www.uniprot.org/) and analyzed using AliView viewer and editing tool [24].

## Results

### Presence of an anti-tau-immunoreactive ∼25 kDa band in rat brain extracts submitted to freeze-and-thaw

**Figure 1:**
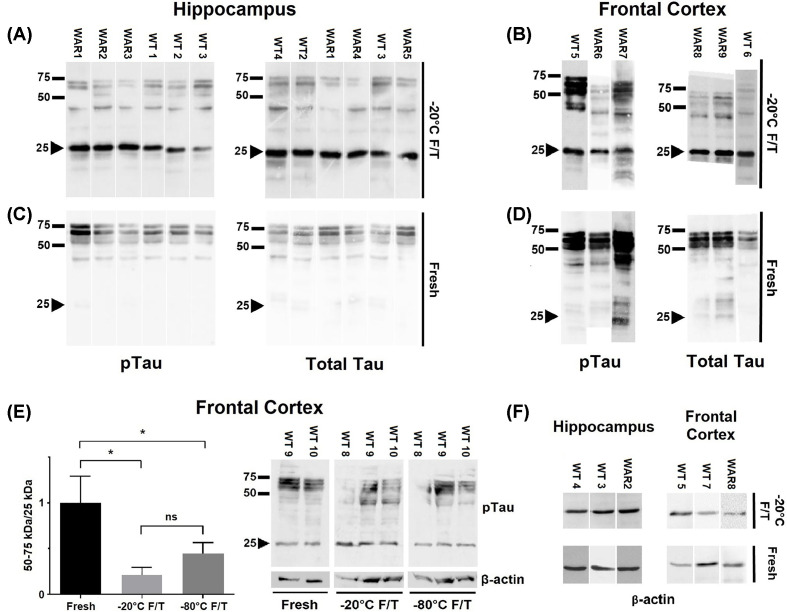
Presence of a ∼25 kDa anti-Tau immunoreactive band in freeze-and-thawed brain extracts of 12-month-old Wistar and WAR rats Extracts were analyzed by WB after a freeze-and-thaw cycle at −20°C (−20°C F/T; **A,B**) or immediately after preparation (fresh; **C,D**). Membranes were probed with either anti-Tau phospho S396 (pTau; A–D,**F**) or anti-Tau [E178] (Total Tau; A–D) antibodies. Extracts were prepared from dorsal hippocampus (A,C) or frontal cortex (B,D) of Wistar (WT) and WAR rats, as discriminated above each lane. (**E**) pTau/25kDa band ratio. A representative WB comparing extracts from frontal cortex stored at different temperatures is shown (*n*=3 per group; *P<0.05). Some extracts were additionally probed for β-actin (F). The arrow-head highlights the ∼25 kDa regions on the membranes.

Elevated Tau phosphorylation levels, particularly in key phosphorylation sites such as Ser^396^, are known to be associated with cognitive impairment and the pathogenesis of several neuropathologies [2,4–7]. With the purpose of investigating Tau hyperphosphorylation in WAR, a rat model of epilepsy and neuropsychiatric comorbidities Cunha et al [25]; Garcia-Cairasco et al [26] including Alzheimer's-associated cognitive decline Alves et al [27], we initially prepared hippocampal extracts from 12-month-old Wistar rats, and evaluated Tau protein levels, both phosphorylated at Ser388 (analogous to Ser396 in humans) and total Tau. In parallel, we determined both total and phospho-Ser388 Tau levels in extracts from 12-month-old WAR animals. The brain extracts had been previously prepared and stored at −20°C. All the comparisons between fresh and freeze/thawed samples were made using the same extracts, and therefore, from the same animals (i.e., the freeze/thawed hippocampal samples loaded and depicted at panel A were aliquoted from the fresh extracts loaded and depicted at panel C; the same for samples depicted at panels B and D). Unexpectedly, in addition to the typical profile of three major bands migrating between 50 and 75 kDa (Figure 1A), which are known to correspond to different isoforms of Tau expressed in rodent brains [28], we found an extra band of ∼25 kDa in all samples probed with either anti-phospho or total Tau antibodies. The ∼25 kDa band was also observed in extracts from frontal cortex stored and handled likewise (Figure 1B).

Zhao et al. [11] have reported the presence of a ∼35 kDa (TCP35) band in transgenic mice brains expressing human Tau, which was correlated with cognitive decline and identified as a proteolytic product of Tau. Motivated by those findings, and considering that freeze-and-thaw may impact the stability of several proteins [29–32], we wondered whether this ∼25 kDa fragment observed under our conditions was endogenously produced or rather corresponded to a product of freeze-and-thaw-induced degradation. Interestingly, when the WB was conducted using fresh samples, the ∼25 kDa band was significantly weaker or even completely absent from both hippocampal (Figure 1C) and frontal cortex (Figure 1D) extracts. Importantly, the bands corresponding to full-length Tau were markedly weaker when the ∼25 kDa band was present, supporting the notion that it represents a degradation product of Tau generated by freeze-and-thawing the extracts. This phenomenon can be clearly observed when fresh and freeze/thawed extracts from frontal cortex of Wistar (WT) rats are probed for pTau in the same membrane (Supplementary Figure S1).

Next, we investigated whether storing the extracts at −80°C, instead of −20°C, would prevent the appearance of the ∼25 kDa band and/or weakening of full-length Tau bands. Data obtained using a different group of animals indicated that storage at −80°C did not prevent the freeze-and-thaw-induced phenomenon, since presence of the ∼25 kDa band and the weakening of 50–75 kDa bands, and consequently the ratio of full-length Tau/25-kDa fragment was similar to that of samples stored at −20°C (Figure 1E). Furthermore, this freeze-and-thaw-induced phenomenon does not seem to equally affect different proteins in the extracts, since no differences in integrity of β-actin between fresh and freeze/thaw extracts were observed (Figure 1E,F). The appearance of the ∼25 kDa fragment, which originated from freeze-and-thawing the brain extracts, was detected at similar degrees in extracts of both Wistar and WAR strains, indicating that this phenomenon is rat strain-independent.

### Identification of the ∼25 kDa band associated with freeze-and-thaw as a degradation product of tau

**Figure 2:**
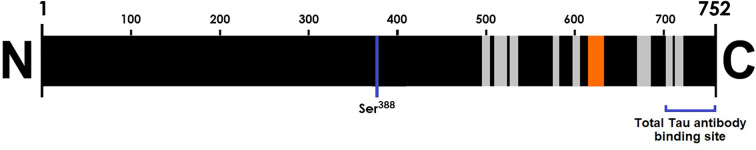
Localization of Tau-derived peptides identified by MS in full-length Tau sequence All the peptides identified in the MS fragment analysis are shown in gray (a unique peptide is highlighted in orange). All the tryptic peptides are located after the Ser^388^ (human analog of human Ser^396^) and near to the C-terminus of full-length Tau from rat. The total Tau antibody binding site, indicated in the C-terminus, refers to the region where the proprietary sequence is located.

In order to verify whether freeze-and-thaw caused Tau fragmentation, we aimed to biochemically identify the freeze-and-thaw-induced ∼25 kDa species by mass spectrometry (MS). For this purpose, an extract of hippocampus from a WAR stored at −20°C was thawed and submitted to protein separation by SDS/PAGE. A piece of the gel in the range of 25 kDa was cut off and subjected to trypsin digestion. The eluted peptides were analyzed by target protein MS. Nine peptides of full-length rat Tau sequence were identified, one of those being a unique peptide (a peptide that exists only in one protein of a proteome of interest, regardless of its length). (Table 1). This result confirmed that the ∼25 kDa band showing immunoreactivity to anti-Tau antibodies is indeed a degradation product of Tau. All of the Tau-associated peptides identified were located near the C-terminus (Figure 2).

**Table 1:** Tryptic peptides identified by targeted MS

Peptide sequence	First–Last amino acid residue
TPPGSGEPPK	491–500
SGYSSPGSPGTPGSR	505–519
TPSLPTPPTR	522–531
IGSTENLK	570–577
LDLSNVQSK	592–600
**HVPGGGSVHIVYKPVDLSK**	609–627
IGSLDNITHVPGGGNK	664–679
TDHGAEIVYK	696–705
SPVVSGDTSPR	706–716

Bold text indicates a unique peptide.

Considering the canonical sequence of rat Tau protein (uniprot.org/uniprot/P19332), and the immunoreactivity of the ∼25 kDa fragment to anti-Tau phospho S396 antibody (the corresponding residue to human Tau Ser^396^ in rats being Ser^388^), we mapped the cleavage site associated with the generation of the 25-kDa fragment to be in between the N-terminus and the Ser^388^. Moreover, the anti-total Tau E178 antibody — which was raised against an antigen situated somewhere between human Tau amino acid 700 to the C-terminus — used in the WB analyses also reassures the truncated portion contains the C-terminus of Tau protein.

### Differences in the generation of the ∼25 kDa Tau fragment in samples from a mice brain disease model and human samples

**Figure 3:**
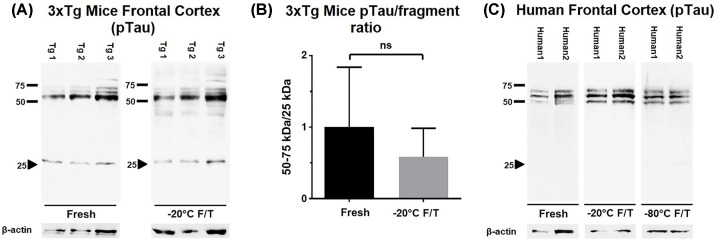
Evaluation of freeze-and-thaw-induced Tau degradation in extracts from experimental models of neurodegeneration (**A**) Representative WB images of 3xTg mice frontal cortex extracts probed for pTau and β-actin. Extracts were either analyzed fresh or after storage at −20°C F/T. The arrow-head indicates the presence of a band near 25 kDa in both fresh and −20°C F/T. (**B**) Full length/fragment ratio obtained from the quantification of 3xTg frontal cortex WB analysis (*n*=6 for each group; *t* test, *P*<0.05). (**C**) WB analysis using human brain slices extracts obtained from two donors.

Tau dysregulation is largely involved in neurodegenerative processes [3,8,9], and more recently, Tau fragmentation has been associated with cognitive decline in mice models of Alzheimer’s disease [11]. After identifying the 25 kDa species as a degradation product of Tau, we wondered whether this phenomenon observed in rat brain samples, including the seizure-prone WAR rat strain, would also take place in samples from a different rodent experimental model of neuropathology. For this purpose, we used frontal cortex extracts from 3xTg-AD mice — a widely used animal model of Alzheimer’s disease [19]. Similar to the observed 25 kDa species in samples from rats, the extracts from 3xTg mice brain presented Tau degradation, i.e. the appearance of a ∼25 kDa fragment along with a slight reduction in bands corresponding to full-length Tau (Figure 3A), which led to a 42% reduction in the ratio between full-length Tau/25 kDa fragment levels (Figure 3B). However, in this cohort this reduction reached no statistical significance.

We also tested a non-rodent experimental model, slice cultures from adult human brains [20]. Surprisingly, in this case, the ∼25 kDa fragment was not observed for fresh samples neither for −20°C F/T, although a faint band showed up in one of the samples stored at −80°C, which seems to be similar to the ∼25 kDa fragment we detected for other species (Figure 3C), suggesting that freeze and thaw induced Tau degradation cannot be completely ruled out even for human samples. A possible explanation for the differences observed in Tau degradation extension between rats, transgenic mice and human samples could be the differences in the primary sequences of Tau between the species, with likely implications to 3D structure, and thus to proteolysis propensity.

**Figure 4:**
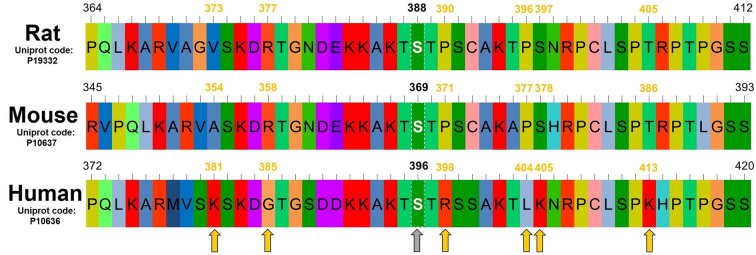
Sequence comparison of Tau residues around phospho Ser^396^ epitope in rodents and humans Comparison between Tau sequences from rat, mouse and human around the serine-phosphorylation site (gray arrow) probed by the anti-Tau phospho S396 antibody (Ser^396^ analog in rat is Ser^388^; in mouse is S^369^). Considering the differences in reactivity and biochemical nature of side chains, the positions where significant substitutions are present comparing rodent *versus* human sequences are indicated by yellow arrows. The default color scheme of AliView was used in the alignment.

In order to estimate the possible contribution of amino acid residue differences to Tau stability in the Tau primary sequence between species, we performed a comparison of the canonical sequences of Tau from rat, mouse and human (Uniprot code: P19332, P10637 and P10636, respectively) using AliView [24]. The sequence alignment comparison exhibits highly conserved sequences around the serine residue targeted by the anti-Tau phospho S^396^ antibody (Figure 4). Despite the similarities, six non-conservative changes between rodents and human sequences were found: a residue with a hydrophobic side chain (V^373^_rat_; A^354^_mouse_) was substituted by a lysine (K^381^_human_), an arginine (R^377^_rat_; R^357^_mouse_) residue was substituted by a glycine (G^385^_human_), a proline (P^390^_rat_; P^371^_mouse_) was substituted by an arginine (R^398^_human_), a proline (P^396^_rat_; P^377^_mouse_) was substituted by a leucine (L^404^_human_), a serine (S^397^_rat_; S^378^_mouse_) residue was substituted by a lysine (K^405^_human_) and a threonine (T^405^_rat_; T^386^_mouse_) was substituted by a lysine (K^413^_human_). Taking the substantial differences regarding the charge, polarity or structure flexibility assigned by those residues into account, it is possible to infer that those differences may play a key role in the proteolysis propensity seen in rodent Tau proteins.

**Figure 5:**
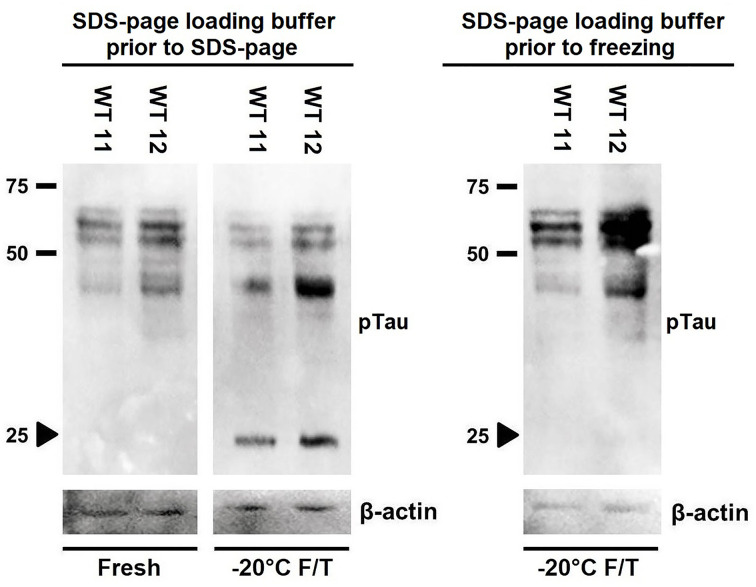
Freeze-and-thaw-induced Tau fragmentation in rat brain extracts is diminished when extracts are frozen in SDS/PAGE loading buffer Representative Western blot of fresh and freeze/thawed extracts from frontal cortex of Wistar (WT) rats probed for pTau. Where indicated, extracts were diluted in SDS/PAGE loading buffer and boiled prior to freezing. Membranes were additionally probed for β-actin.

As a possible strategy to minimize Tau fragmentation induced by freeze/thaw in brain extracts, we investigated the impact of adding SDS/PAGE loading buffer and boiling before freezing the extracts on Tau integrity. Interestingly, we found that denaturation induced by SDS/PAGE loading buffer and boiling significantly prevented freeze/thaw-induced Tau degradation, as indicated by both the preservation of full-length Tau bands and the absence of the 25 kDa band, as seen for the fresh extracts (Figure 5). Possible explanations for the protective effects of this procedure are the elimination of a proteolysis-prone site in Tau due to a conformational change; or the inactivation of one or more proteases (resistant to the inhibitors added to the buffer used to prepare the brain extracts) involved in Tau degradation.

## Discussion

In this work we present results indicating that a ∼25 kDa fragment generated by Tau degradation is present when extracts from rat brains are subjected to freeze and thaw — a common step prior to WB analyzes. Although reports of protein degradation in brain samples stored as frozen extracts have been shown for samples stored in the absence of protease inhibitors [33], our present study shows the occurrence of proteolysis even in extracts prepared using a protease inhibitor cocktail. Surprisingly, this phenomenon was minor in 3xTg mice brains, an animal model of neurodegeneration, and practically absent from samples from human brains, suggesting that differences in primary sequences of Tau influence the liability of this protein to freeze-and-thaw-induced proteolysis. Considering the primary sequence of human Tau shows more positively charged residues compared with the rodent sequences (Figure 4), it is conceivable that a slight hydrophobicity increase in rodent Tau protein may account for a more compact, water-protected conformation compared with human Tau. This could result in an increased susceptibility of this region to freeze/thaw-induced partial denaturation and consequent proteolysis.

Given the widespread use of WB to determine Tau levels in brain extracts in experimental models of neurodegeneration, it is reasonable to raise a concern on the possible impact of freeze-and-thaw-induced Tau proteolysis on the reliability of reported findings in which rodent Tau levels have been determined by WB. The emergence of the 25 kDa band, concomitant to the weakening of the bands corresponding to full-length Tau (which are used to quantify the levels of Tau) might therefore introduce an artifactual outcome. It is not possible to completely rule out that Tau fragmentation is diminished or even absent in brain extracts subjected to short term storage (e.g., 24 h) at either −20 or −80°C. Although this is a relevant point, we believe this condition correspond to a minority of the cases when thawing a frozen extract is needed.

Among the identified tryptic peptides, it is interesting to notice a lack of peptides located in the near vicinity of residue Ser^388^, the epitope of the pTau antibody used in the WB analysis. This may be due both by Ser^388^ phosphorylation, which impairs peptide identification due to the mass change caused by the phosphorylation, and the small size of tryptic peptides originated from this region, which fall off the mass range of the mass spectrometer used (∼800 to 2000 Da). Further analysis will be necessary to investigate in detail the proportions of Tau-derived peptides in freeze-and-thaw Tau extracts.

Assuming the freeze and thaw induced proteolysis occurs near Ser^388^ (the analog residue of human Ser^396^ in Tau sequence from rats), the predicted molecular weight of the C-terminal fragment is ∼35 kDa. Although the reason for the ∼10 kDa discrepancy between the apparent MW we have observed experimentally and the predicted molecular weight is not clear, it is well known that several proteins present anomalous behavior in MW determination by SDS/PAGE (e.g. [34]). It is important to consider that Tau mRNA alternative splicing generates several isoforms of rat Tau (uniprot.org/uniprot/P19332#cross_references), and this may implicate in alternative Tau isoforms with unpredicted MW [35]. Another point to be elucidated is whether Tau protein has an intrinsic propensity to freeze-and-thaw-induced degradation or instead other factors are required to trigger it. Thus, further analyses are necessary to, firstly, determine which isoforms undergo proteolysis induced by freeze-and-thaw to generate the ∼25 kDa fragment detected in our experiments and secondly, to evaluate whether external factors are in fact required for Tau degradation to occur. To address these issues, we intend to perform experiments using purified proteins in the future, in order to analyze whether these behave similarly to the endogenous protein evaluated in the present study.

Interestingly, Zhao et al. (2016) have recently mapped a proteolysis site for caspase in the human Tau isoform 0N4R (amino acid sequence: HVPGGGSVQIVYKPVD), which generates a 35-kDa Tau fragment correlated with cognitive impairment in mice and humans presenting tauopathies [11]. Although this region is partially conserved and present in all isoforms of rat Tau (uniprot.org/uniprot/P19332), it does not seem to correlate with the alleged cleavage region hypothesized in the present study, since the caspase site is located approximately 200 residues apart from the Ser^388^ residue immunodetected in the 25-kDa fragment described in this work. More studies are necessary in order to map the exact proteolysis site associated with the cleavage we have detected in the present study. Moreover, the conformational flexibility of the region surrounding residue Ser^396^ of human Tau has been recently demonstrated by the Ser^396^ phosphorylation-induced α-helix to β-sheet conversion, with possible implications to microtubule binding and aggregation [36].

The importance of these findings is reinforced when we take into account that the freeze and thaw induced Tau proteolysis was observed in samples from both wildtype (Wistar) and WAR rats, the later a strain that has been used to model epilepsy and associated comorbidities [26]. Importantly, WAR animals have also recently shown to present spatial memory and learning deficits that correlate with increased Tau phosphorylation in hippocampus [27,37]. Moreover, phosphorylated Tau levels are widely used as an indicator of neurodegeneration, in particular the phosphorylated epitope evaluated in this work (Ser^396^ in human sequence), which is implicated in the pathophysiology of several neurological diseases such as Alzheimer’s and Pick disease [2–6]. Therefore, it is not possible to rule out that, at least in some studies analyzing rodent Tau levels by WB (e.g. [38–40]), the reported quantification may have been impacted by the sample storage process, notably freeze and thawing brain extracts. Unfortunately, in many studies reporting Tau level analysis by WB, no detailed description of sample handling prior to WB is depicted. In addition, the representative images presented in many works (e.g. [41–45]) usually do not allow a comprehensive evaluation on the presence of Tau fragments, due to membrane image cutting.

In summary, we report that freeze-and-thawing rat brain extracts led to Tau protein degradation, detected by the presence of an extra band of ∼25 kDa, simultaneously to weakening of the three major bands corresponding to full-length Tau in WB analysis. Based on this observation, we strongly recommend that analysis of Tau levels in rodent brain samples be performed using fresh extracts or, alternatively, extracts stored at −20°C which have been previously diluted in SDS/PAGE loading buffer and boiled, the later found to be a simple and effective strategy to prevent freeze-and-thaw-induced Tau degradation. Considering the wide use of WB to evaluate neurodegeneration in tauopathies, we anticipate that following this recommendation will lead to more accurate interpretations in future studies on Tau-related disorders.

## Supplementary Material

Supplementary Figures S1-S9 and Table S1

## Data Availability

The authors confirm that the data supporting the findings of the present study are available within the article and its supplementary materials. The raw data underlying the WB results reported are presented in the Supplementary Material.

## Abbreviations

AD: Alzheimer's disease
MAP: Microtubule-associated protein
MS: Mass spectrometry
TBS: Tris-buffered saline
USP: University of São Paulo
WAR: Wistar Audiogenic Rat
WB: Western blotting
