# Supplementary material for: A freeze-and-thaw-induced fragment of the microtubule-associated protein tau in rat brain extracts: implications for the biochemical assessment of neurotoxicity
Source: Biosci Rep. 2021 Mar 24;41(3):BSR20203980. doi: 10.1042/BSR20203980 (PMC7990086; doi:10.1042/BSR20203980)
Supplement: Supplementary Figures S1-S9 and Table S1 [file BSR-2020-3980_supp.pdf]

SUPPLEMENTARY MATERIAL

Figure S1. Raw data of **Fig.1 – Panel A** western blotting of -20°C freeze/thawed rat hippocampus extracts (lanes used in the main figure are indicated by dotted frames).

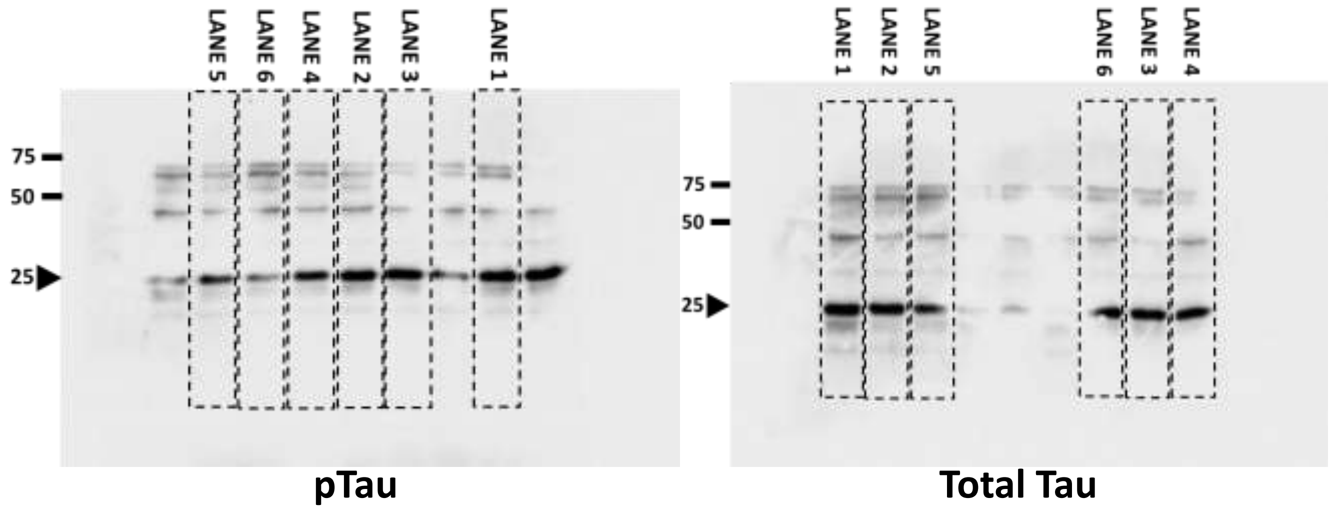

Figure S2. Raw data of **Fig.1 – Panel B** western blotting of -20°C freeze/thawed frontal rat cortex extracts (lanes used in the main figure are indicated by dotted frames).

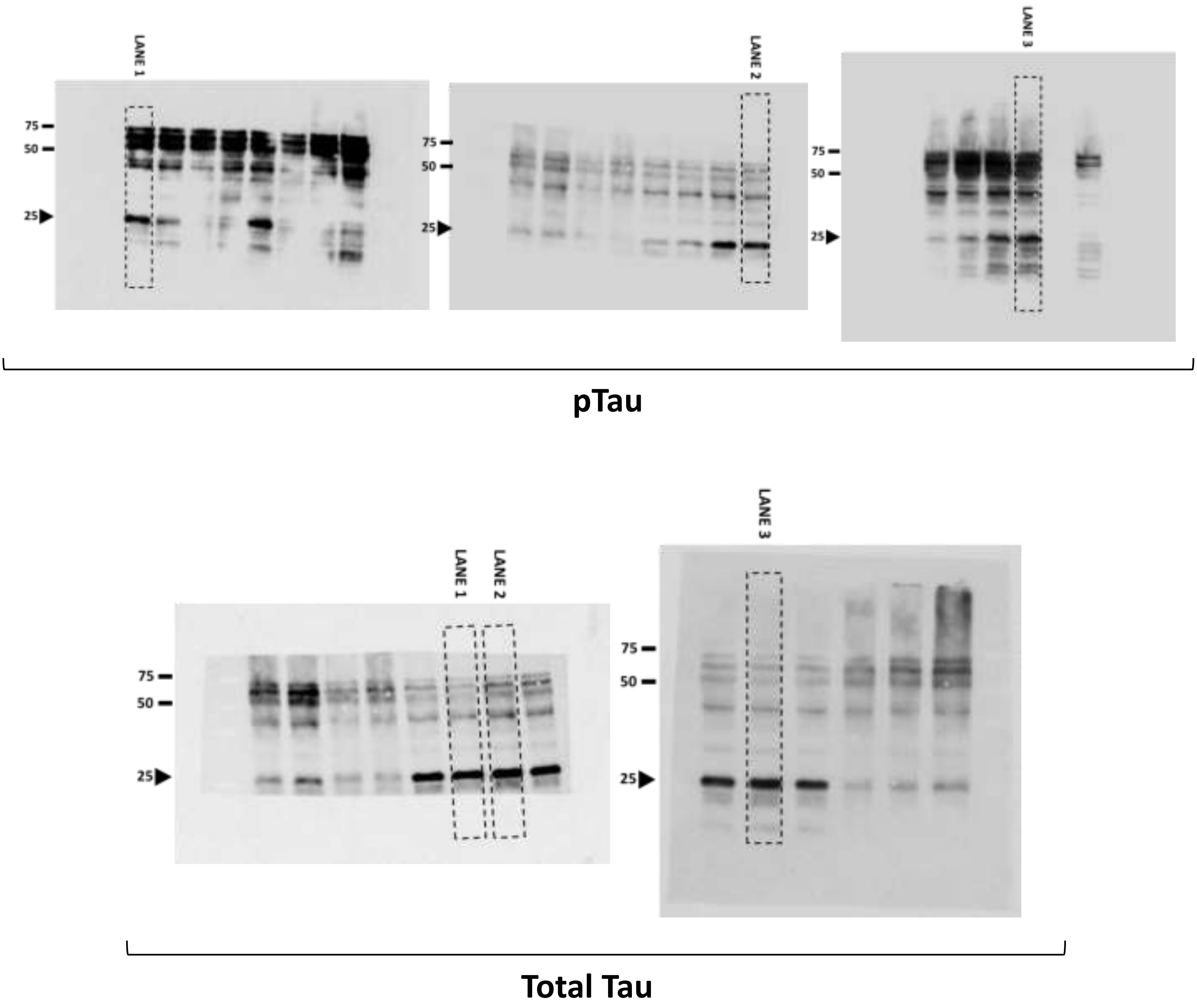

Figure S3. Raw data of **Fig.1 – Panel C** western blotting of fresh rat hippocampus extracts (lanes used in the main figure are indicated by dotted frames).

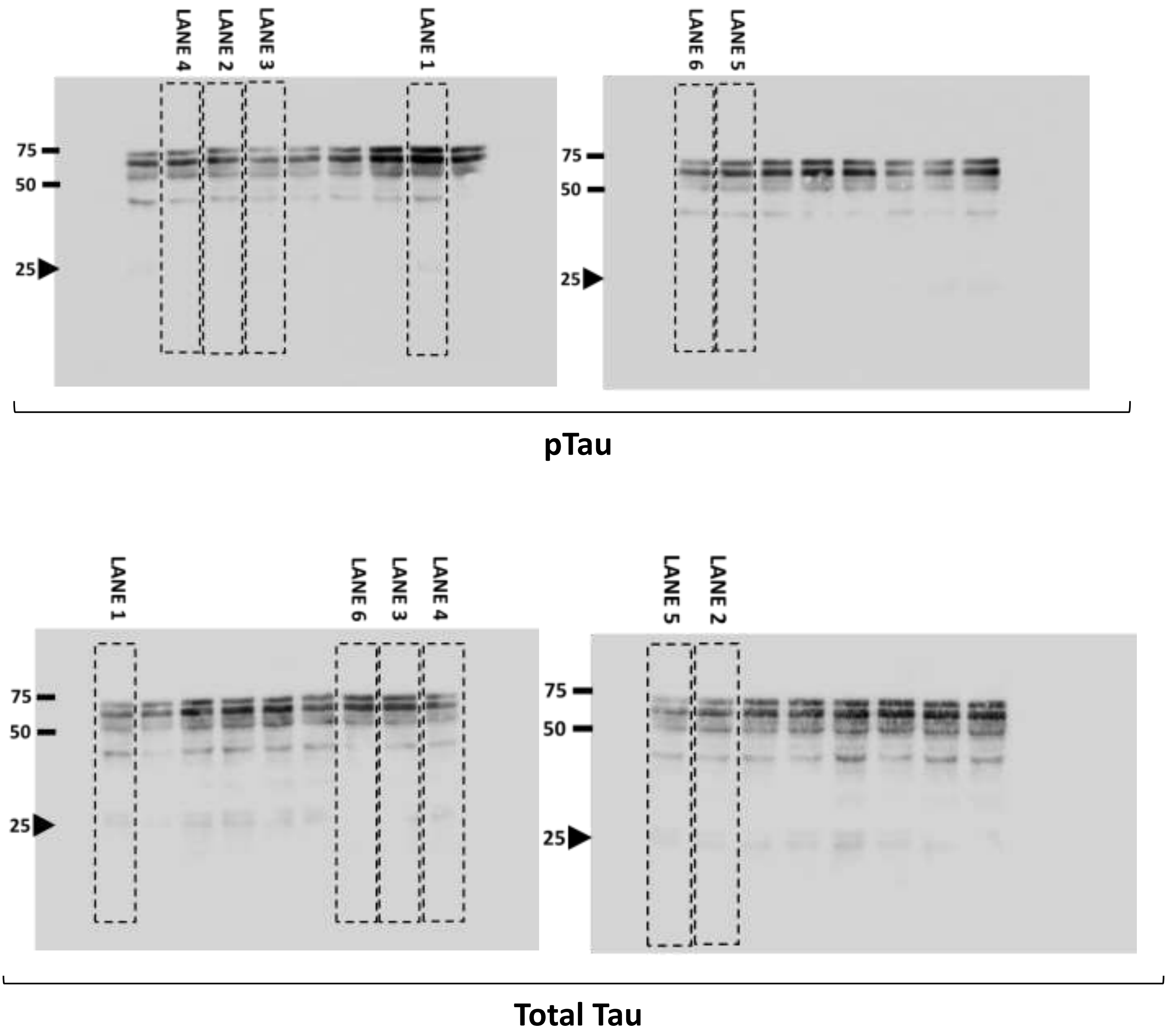

Figure S4. Raw data of **Fig.1 – Panel D** western blotting of fresh rat frontal cortex extracts (lanes used in the main figure are indicated by dotted frames).

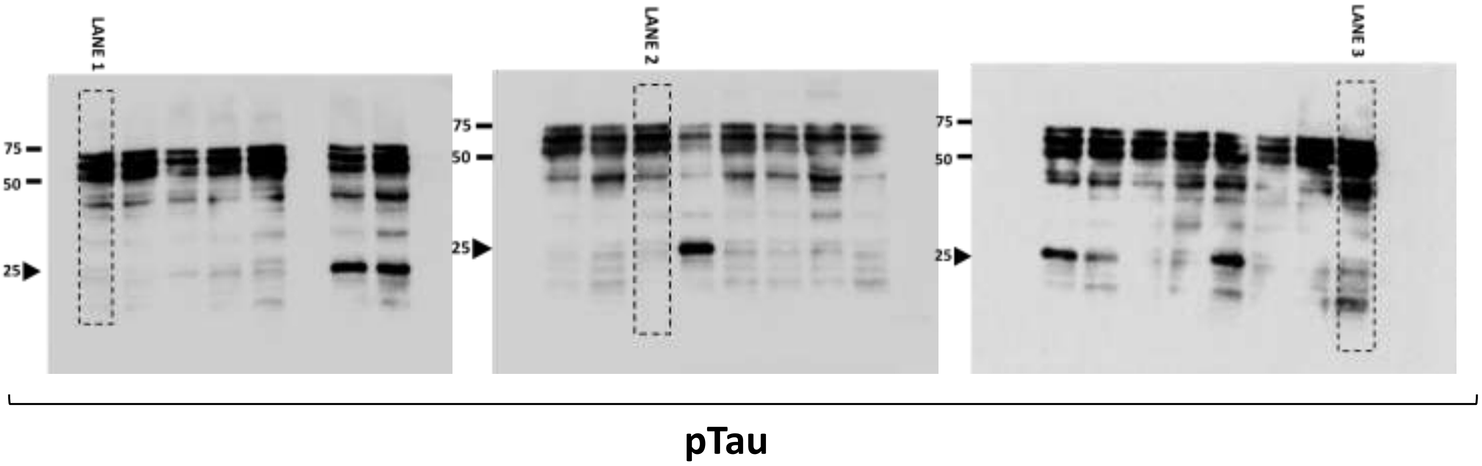

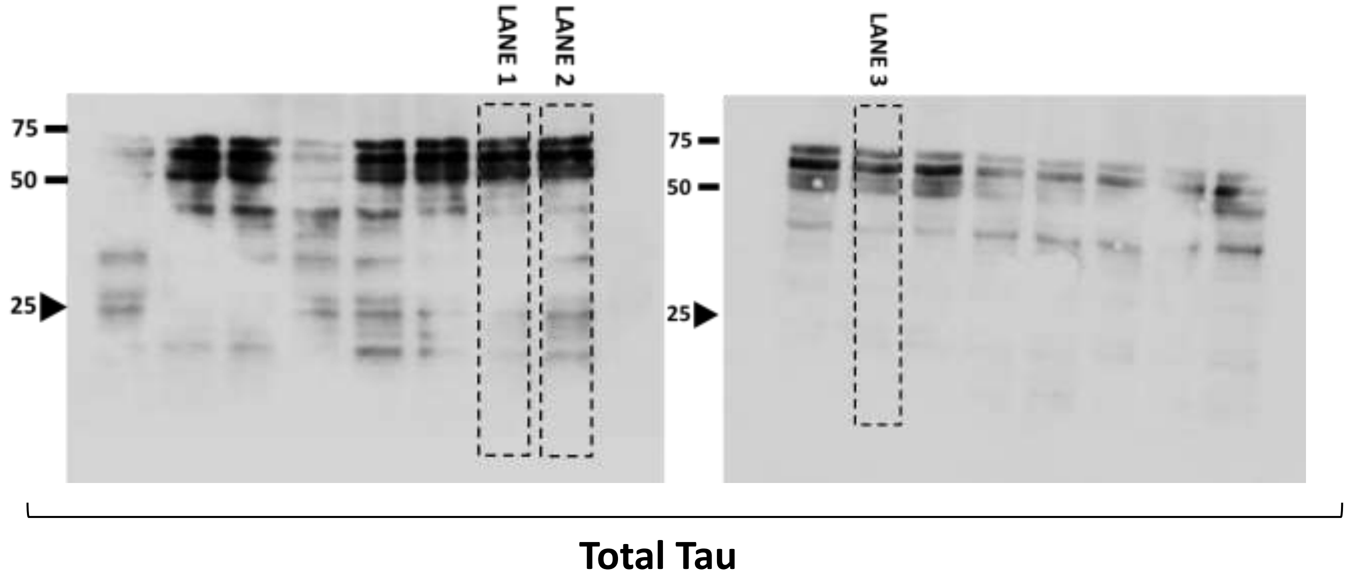

Figure S5. Raw data of **Fig.1 – Panel E** western blotting of freeze/thawed rat frontal cortex extracts (lanes used in the main figure are indicated by dotted frames). Asterisks (*) stand for lanes with rat extracts from other cerebral tissue.

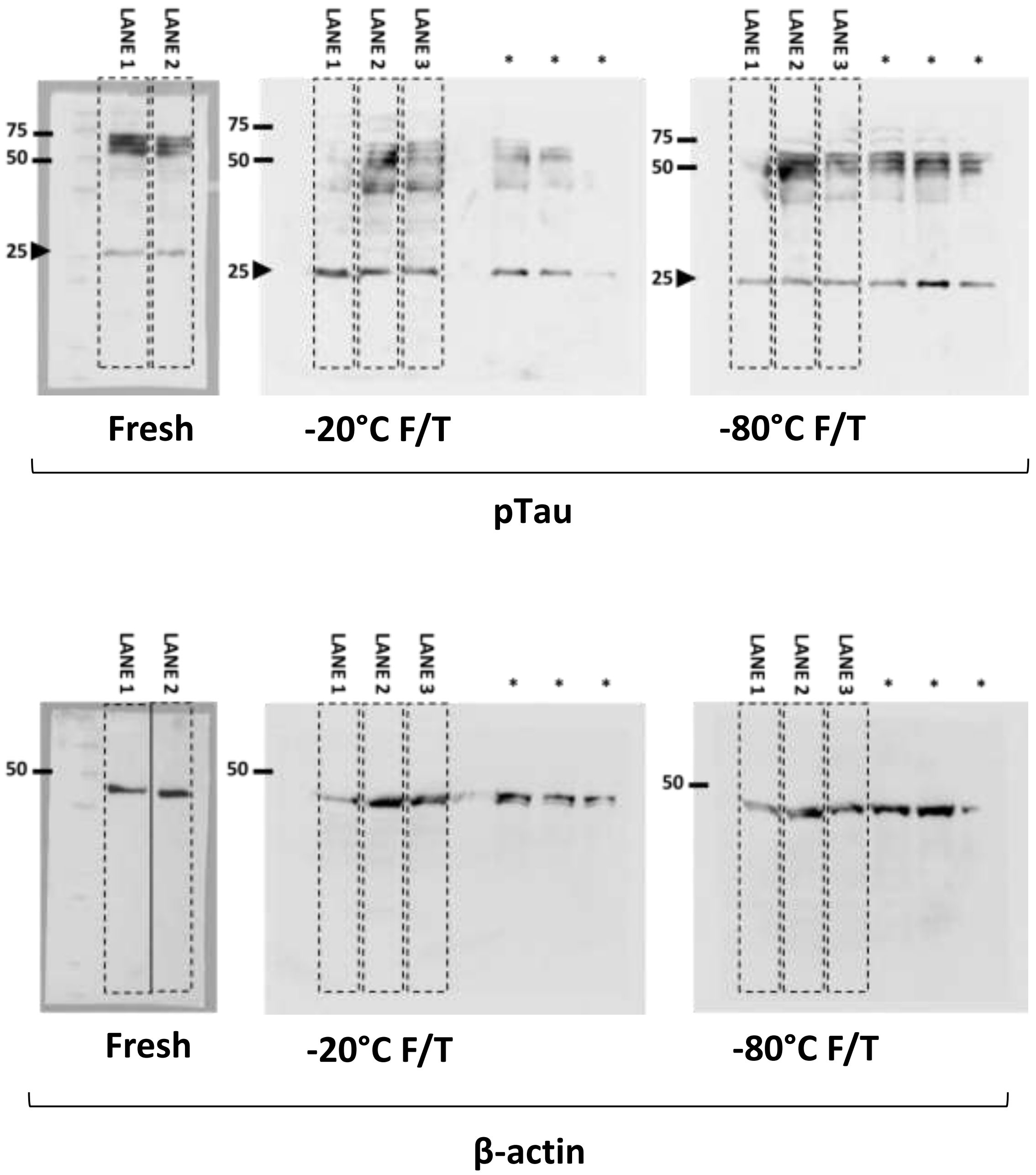

Figure S6. Raw data of **Fig.1 – Panel F** western blotting of fresh and freeze/thawed extracts from rat hippocampus and frontal cortex (lanes used in the main figure are indicated by dotted frames).

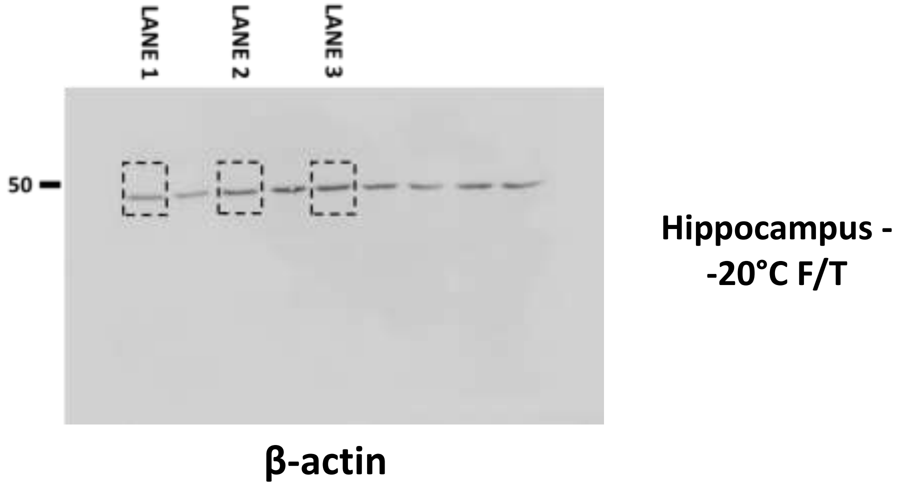

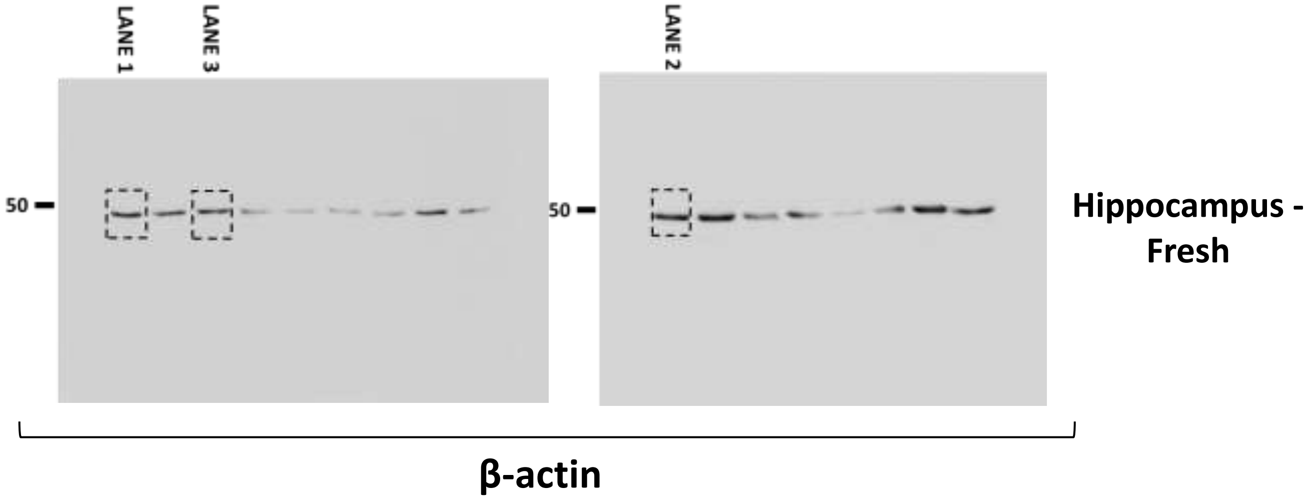

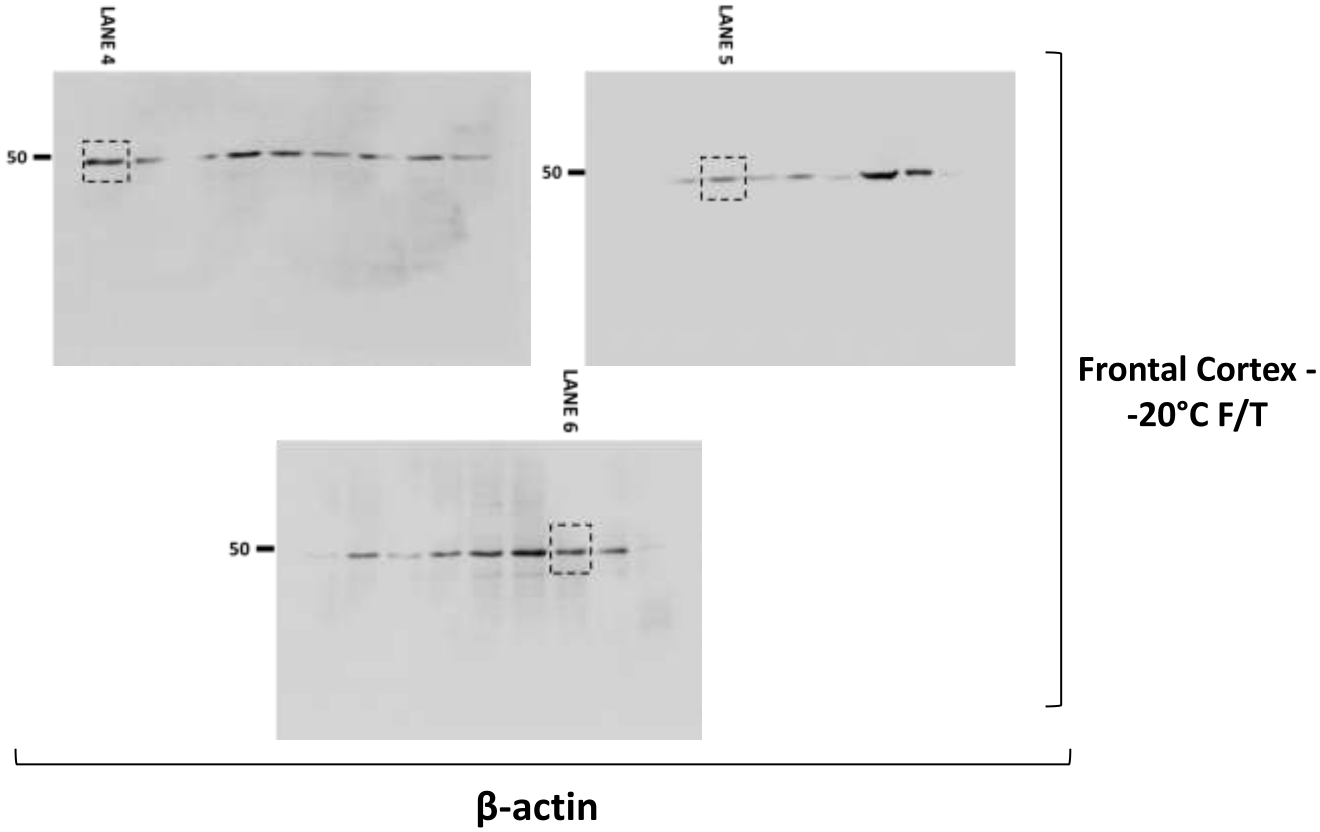
LANE 4
LANE 5
LANE 6
50
50
50
Frontal Cortex - -20°C F/T
β-actin

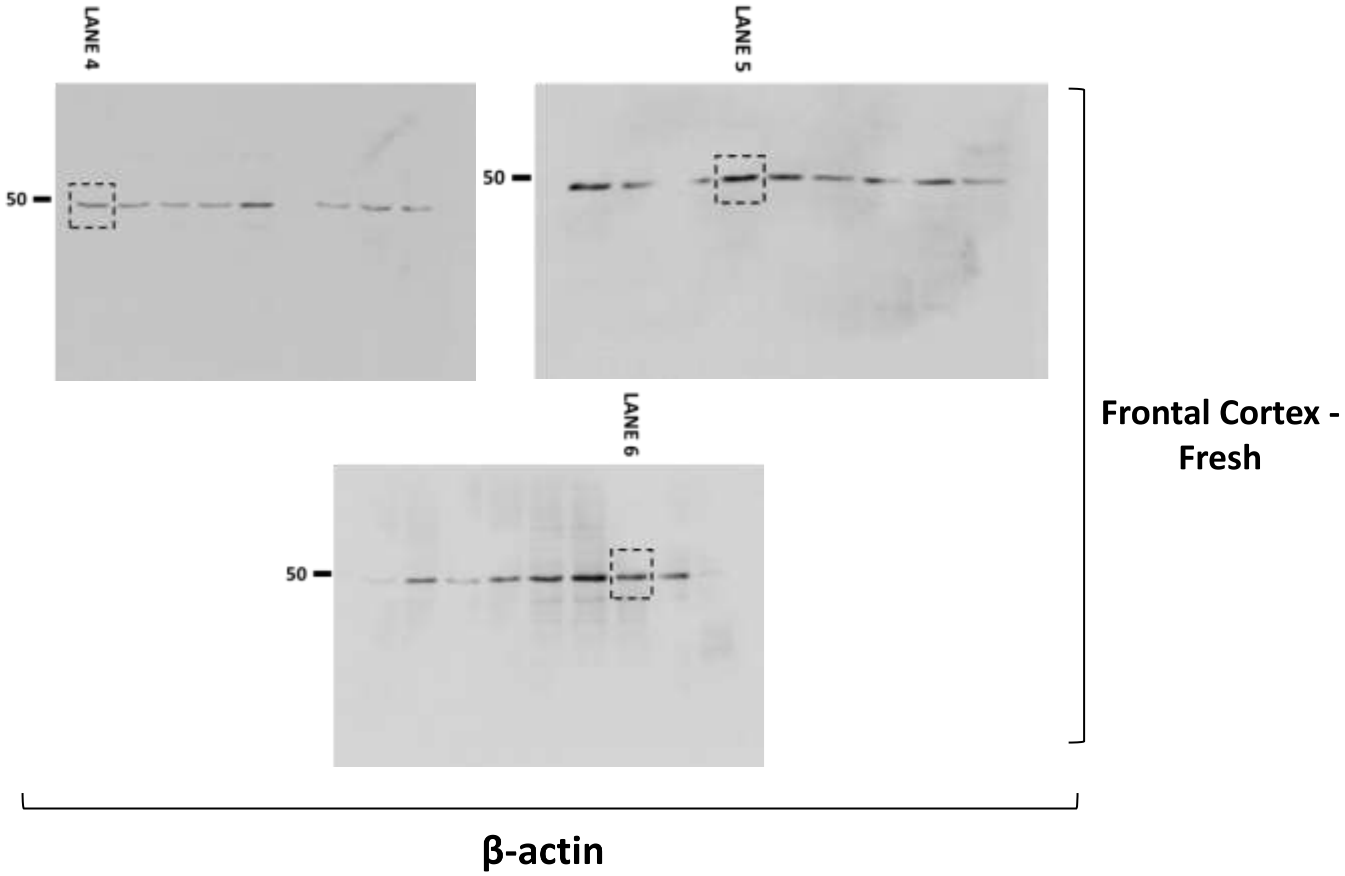
LANE 4
LANE 5
LANE 6
50
50
50
Frontal Cortex - Fresh
β-actin

Figure S7. Raw data of **Fig.3 – Panel A** western blotting of fresh and freeze/thawed extracts from 3xTg mice frontal cortex (lanes used in the main figure are indicated by dotted frames).

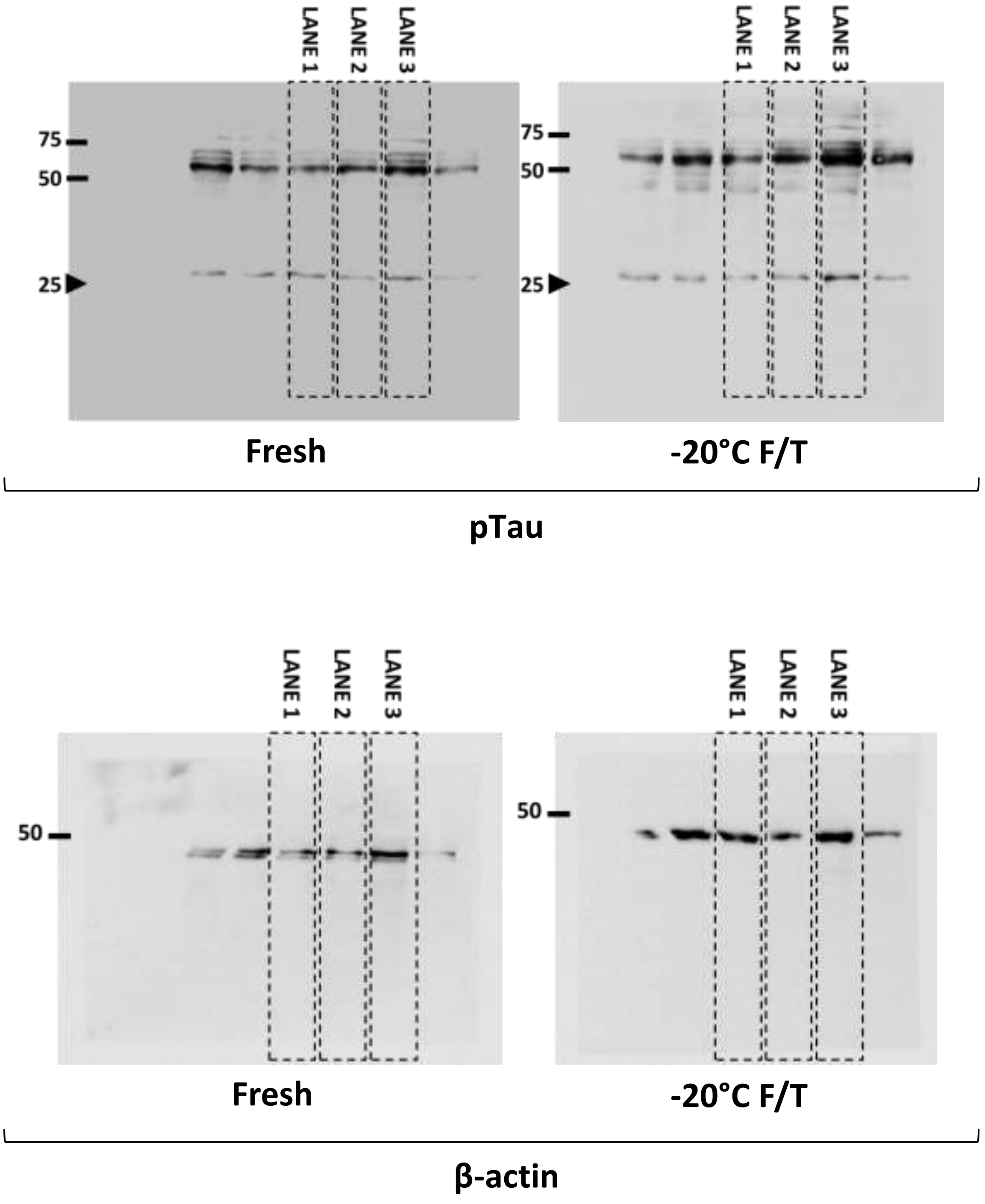

Figure S8. Raw data of **Fig.3 – Panel C** western blotting of fresh and freeze/thawed extracts from human frontal cortex (lanes used in the main figure are indicated by dotted frames). Asterisks (*) stand for lanes with human extracts frozen at -80°C.

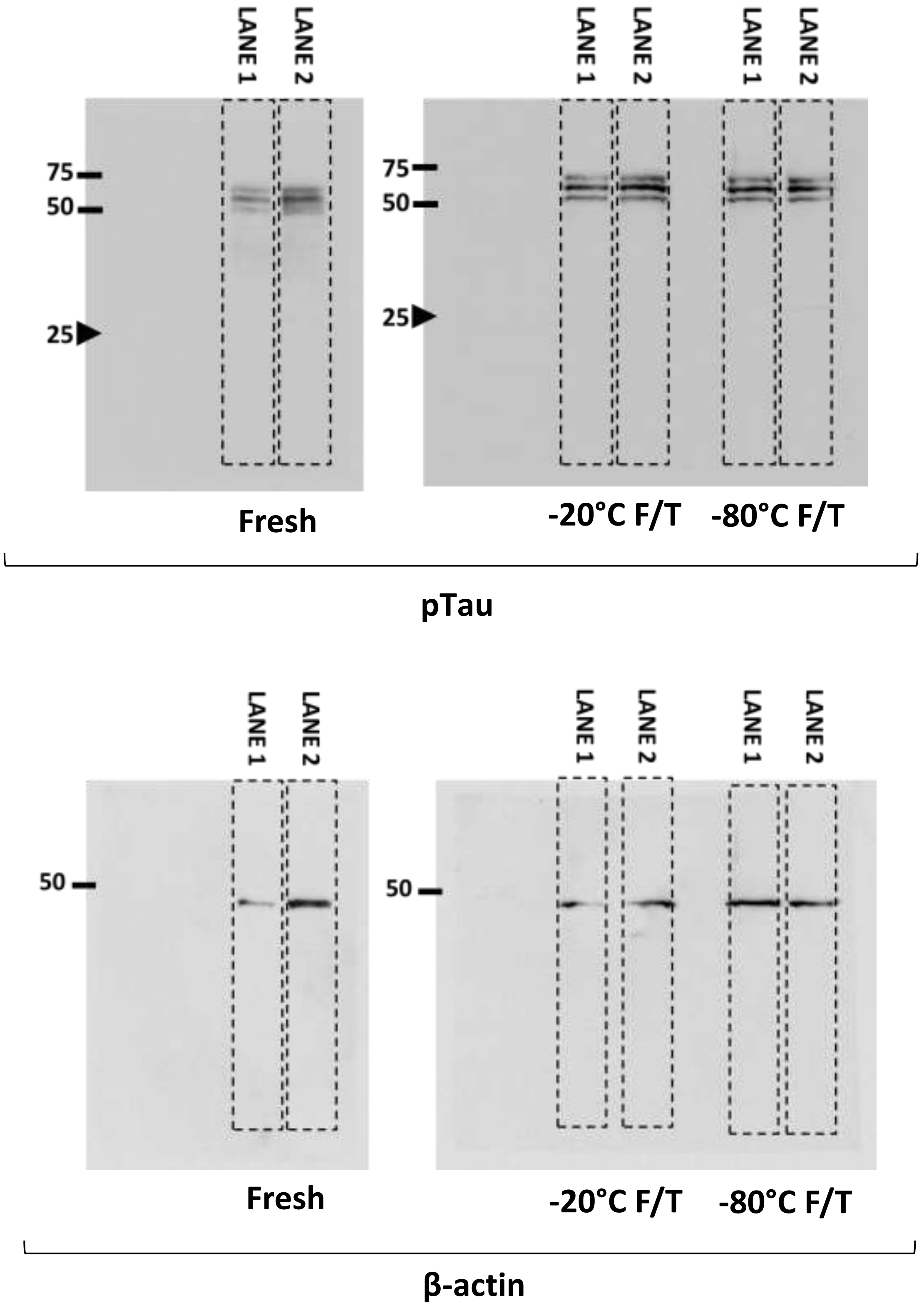

Figure S9. Raw data of **Fig.5 – Panel A** western blotting of fresh, freeze/thawed and pre-treated (previously diluted in loading buffer prior to freezing) freeze/thawed extracts from frontal cortex of Wistar (WT) rats, probed in the same membrane for pTau and β-actin.

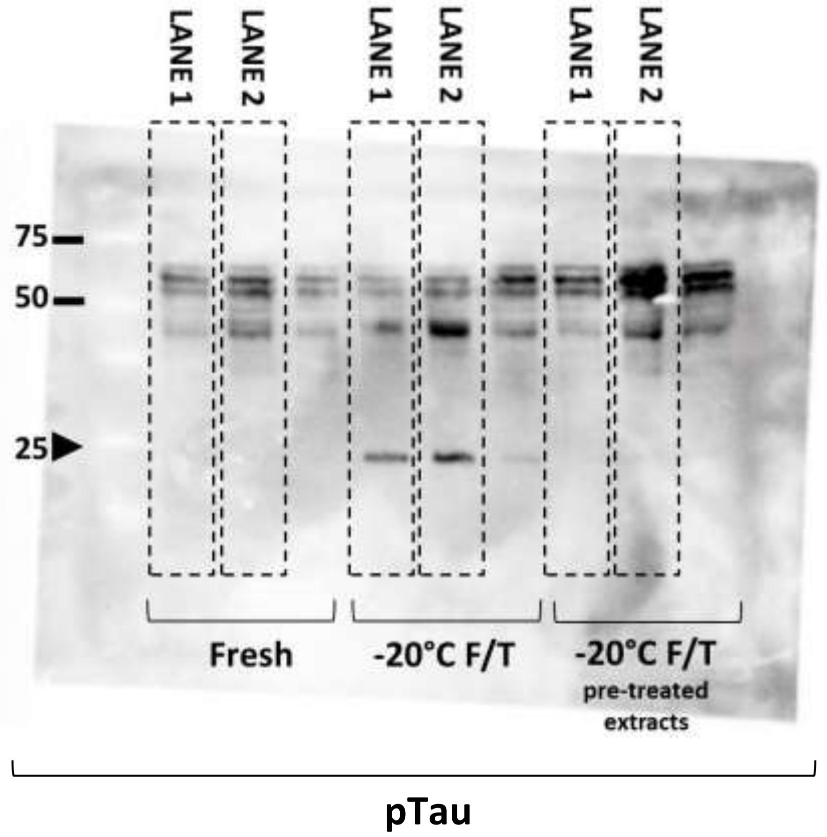

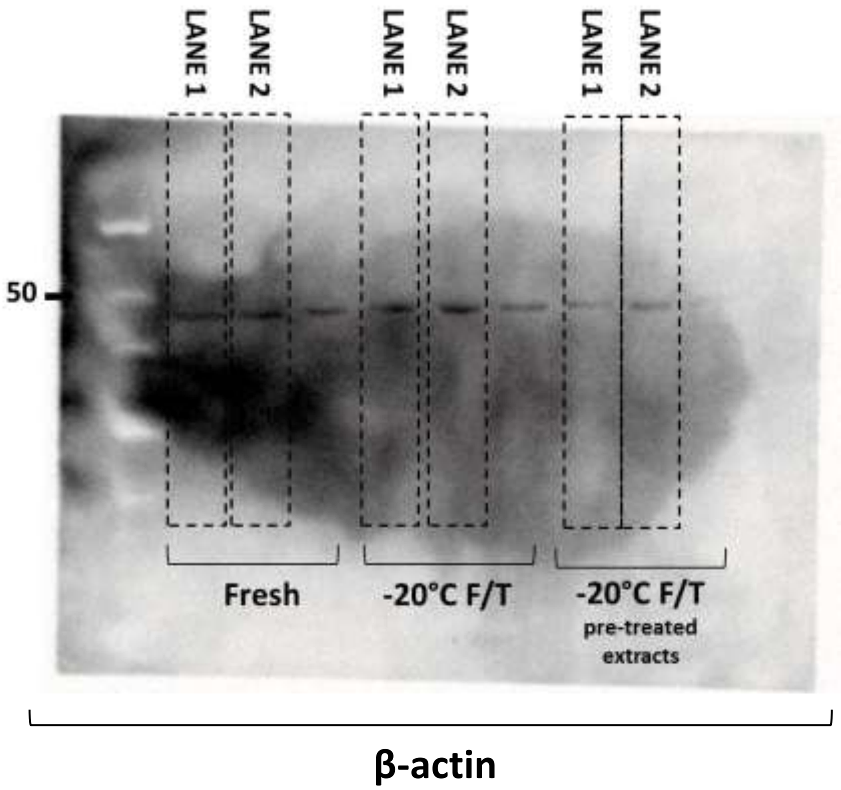

Table S1. Storage time of each freeze/thawed extract used in western blotting analysis displayed at **Fig.1, Fig.3** and **Fig.5**.

Figure	Panel	Sample	Evaluated epitope	Storage time (-20°C or -80° C)
1	A	WAR 1	pTau	35 days
1	A	WAR 2	pTau	35 days
1	A	WAR 3	pTau	35 days
1	A	WT 1	pTau	35 days
1	A	WT 2	pTau	35 days
1	A	WT 3	pTau	35 days
1	A	WT 4	Total Tau	35 days
1	A	WT 2	Total Tau	35 days
1	A	WAR 1	Total Tau	35 days
1	A	WAR 4	Total Tau	35 days
1	A	WT 3	Total Tau	35 days
1	A	WAR 5	Total Tau	35 days
1	B	WT 5	pTau	undetermined (≥ 8days)
1	B	WAR 6	pTau	4 months
1	B	WAR 7	pTau	15 days
1	B	WAR 8	Total Tau	4 months
1	B	WAR 9	Total Tau	4 months
1	B	WT 6	Total Tau	9 months
1	E	WT 8	pTau/β-actin	15 days
1	E	WT 9	pTau/β-actin	15 days
1	E	WT 10	pTau/β-actin	15 days
1	F	WT 4	β-actin	35 days
1	F	WT 3	β-actin	35 days
1	F	WAR 2	β-actin	35 days
1	F	WT 5	β-actin	undetermined (≥ 8days)
1	F	WT 7	β-actin	15 days

1	F	WAR 8	β-actin	4 months
3	A	3xTg 1	pTau/β-actin	15 days
3	A	3xTg 2	pTau/β-actin	15 days
3	A	3xTg 3	pTau/β-actin	15 days
3	C	Human 1	pTau/β-actin	15 days
3	C	Human 2	pTau/β-actin	15 days
5	-	WT 11	pTau/β-actin	15 days
5	-	WT 12	pTau/β-actin	15 days
